# Total Syntheses of Pladienolide-Derived Spliceosome Modulators

**DOI:** 10.3390/molecules26195938

**Published:** 2021-09-30

**Authors:** Jaehoon Sim, Eunbin Jang, Hyun Jin Kim, Hongjun Jeon

**Affiliations:** 1 College of Pharmacy, Chungnam National University, Daejeon 34134, Korea; dmsqls3747@naver.com; 2 Therapeutics and Biotechnology Division, Korea Research Institute of Chemical Technology, 141 Gajeong-ro, Yuseong-gu, Daejeon 34114, Korea; hyunjin@krict.re.kr

**Keywords:** pladienolide class, spliceosome, total synthesis, natural products, macrocycles

## Abstract

Pladienolides, an emerging class of naturally occurring spliceosome modulators, exhibit interesting structural features, such as highly substituted 12-membered macrocycles and epoxide-containing diene side chains. The potential of pladienolides as anti-cancer agents is confirmed by H3B-8800, a synthetic analog of this natural product class, which is currently under Phase I clinical trials. Since its isolation in 2004 and the first total synthesis in 2007, a dozen total syntheses and synthetic approaches toward the pladienolide class have been reported to date. This review focuses on the eight completed total syntheses of naturally occurring pladienolides or their synthetic analogs, in addition to a synthetic approach to the main framework of the natural product.

## 1. Introduction

Ribonucleic acid (RNA) post-transcriptional modification is a vital biological process in most eukaryotic cells. It allows the production of mature RNA that can perform normal and diverse functions in the cell [1]. One representative process in this modification is RNA splicing. Premature RNA consists of non-coding intron regions and coded exon regions. During RNA splicing, introns are removed, leaving exons that are re-ligated and can function as mature RNA. Because of its significant role in protein production, the mRNA splicing process in cells is tightly regulated. Indeed, splicing defects such as exon-skipping can induce changes in the levels of specific splicing isoforms, causing a variety of diseases, including cancer [2].

The splicing reaction is regulated by a spliceosome, which is a dynamic multimegadalton ribonucleoprotein (RNP) complex composed of five small nuclear RNA ribonucleoproteins (snRNPs: U1, U2, U4, U5, and U6) and numerous proteins [3]. Depending on the function of the spliceosome, a variety of mature mRNAs can be produced from the same pre-mRNA and can be translated to diverse proteins, such as antibodies, in a process called alternative splicing. Alternative splicing is an important mechanism for generating proteomic diversity from a relatively limited number of protein-coding genes [4]. Given the importance of RNA splicing and the fundamental role of the spliceosome in post-transcription, the spliceosome has gained attention as a target for fighting cancer. For example, the splicing factor 3b (SF3b) complex, a representative spliceosomal component, is the most frequently mutated splicing factor in cancers [5] such as myelodysplastic syndromes [6], acute myeloid leukemia (AML) [7], chronic lymphocytic leukemia (CLL) [8], and various solid tumors [9,10,11].

**Figure 1:**
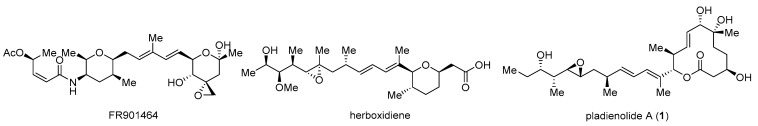
Representative examples of naturally occurring spliceosome modulators.

Most splicing modulators reported to date are naturally occurring molecules, including the FR class [12,13,14,15], herboxidiene class [16,17], and pladienolide class (Figure 1). Among them, pladienolides are structurally unique in that they possess a highly substituted macrocyclic core structure that has captured the attention of the synthetic chemistry community. Pladienolides are naturally occurring macrolides that were first isolated by Eisai Co. in 2004 from Mer-11107, an engineered strain of *Streptomyces platensis* [18,19,20]. This polyketide natural product was later reported to interfere with spliceosome function by targeting the SF3b subunit in a dose-dependent manner [21]. Due to this interesting biological function, several medicinal chemistry efforts have been made using simplified analogs [22,23] or synthetic molecules hybridized with another splicing modulator [24]. In addition, the biosynthetic production of novel pladienolide analogs has been recently reported by means of native expression of a pathway-specific activator [25,26].

Pladienolide B, one of the related macrolides, has been proven to be the most active congener, with nanomolar IC_50_ values against various human cancer cell lines [20,27,28,29]. The cocrystal structure of pladienolide B and the human SF3b core discovered in 2018 confirmed their inhibitory and modulatory effects via splicing [30]. In addition, pladienolide B has been recently reported to prevent SARS-CoV-2 replication at non-toxic concentrations in human cells by targeting the splicing process [31].

**Figure 2:**
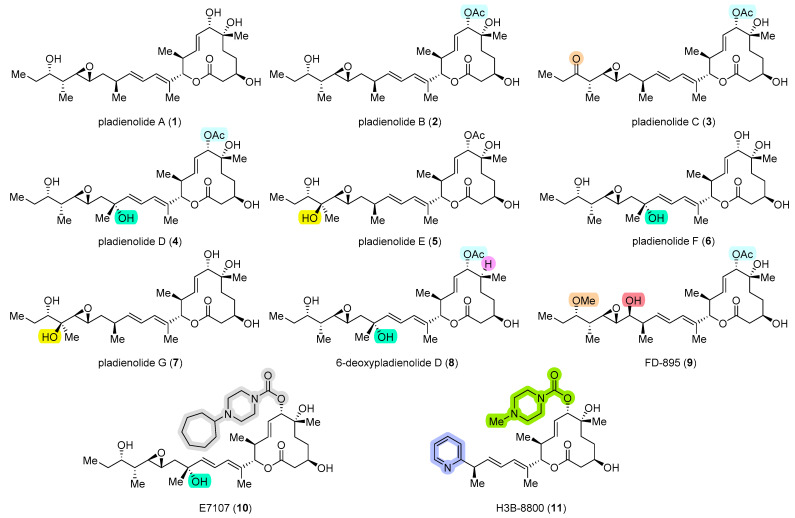
Chemical structure of pladienolides A–G (**1**–**7**), 6-deoxypladienolide D (**8**), FD-895 (**9**), and synthetic analogs (**10** and **11**). The colors show the part of each compound that is structurally different from the structure of pladienolide A (**1**).

There are several natural pladienolide derivatives, including pladienolide A–G (**1**–**7**) [18,19,20], 6-deoxypladienolide D (**8**) [32], and FD-895 (**9**) [33,34], as well as synthetic derivatives E7107 (**10**) and H3B-8800 (**11**) (Figure 2). E7107 (**10**) is an analog of pladienolide D, developed by Eisai Co., and the first SF3B1 modulator that entered phase I clinical trials on patients with different types of solid tumors (NCT00459823 and NCT00499499) [35]. However, the trials were discontinued because of unexpected toxicity at higher doses, resulting in vision loss [36,37,38]. H3B-8800 (**11**), another analog developed by H3 Biomedicine, a subsidiary of Eisai Co., is an orally bioavailable drug currently under phase 1 clinical trials to treat patients with myelodysplastic syndromes, AML, and chronic myelomonocytic leukemia (NCT02841540) [39].

Owing to a growing interest in RNA splicing for drug discovery, a number of studies and patents have been reported, including review articles that (partially) cover the biological features and structure–activity relationship of pladienolides [40,41,42,43,44]. In this review, we provide a comprehensive overview of the total synthesis of pladienolides or their core structures, with a detailed analysis of the synthetic routes. We present a summary of the total syntheses of pladienolides from the time the first synthesis was reported to the present date (2007–2021). This review is divided into two main sections, based on the macrocyclization strategy.

## 2. Synthesis of Pladienolides

### 2.1. Synthetic Strategies Regarding Pladienolides

**Figure 3:**
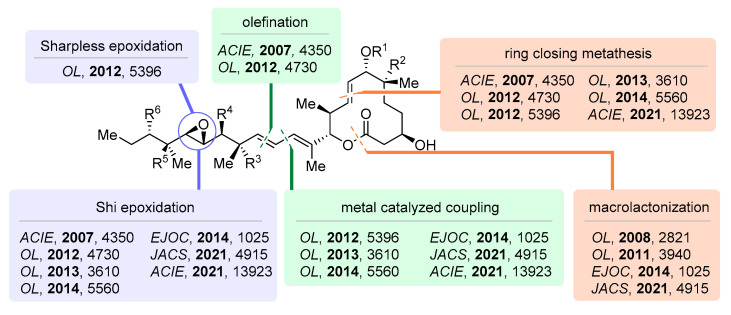
Brief summary of the disconnections of pladienolides as reported, 2007–2021.

Pladienolides are composed of two fragments: a 12-membered macrolactone ring with five stereocenters and a side chain bearing up to six contiguous stereocenters, with a *trans* epoxide moiety at the center (Figure 3). Most of the reported pladienolide syntheses are based on the conjugation between these two fragments via metal-catalyzed coupling or olefination. The major macrolactone moiety has been established by ring-closing metathesis (RCM) or macrolactonization as the key last step. In addition, the absolute stereochemistry of *trans* epoxides has been controlled by external chiral sources, such as AD-mix (Sharpless asymmetric epoxidation) [45] or fructose-derived organocatalysts (Shi epoxidation) [46]. Pladienolides have methyl or hydroxy groups as substituents, the stereocenter of which is often controlled by asymmetric aldol reactions. 

### 2.2. Macrocyclization via Ring-Closing Metathesis

Since the discovery of highly bench-stable and functional-group-tolerant metathesis catalysts, cross-metathesis has been considered a powerful tool for natural product synthesis [47]. In particular, RCM has become the most popular approach for synthetically challenging medium and macrocyclic natural products [48]. Unsurprisingly, a number of synthetic strategies for 12-membered core scaffold of pladienolides rely on RCM, including those published by Kotake [49], Ghosh [50], Burkart [34], Chandrasekhar [51], Keaney [52], Krische [53], and their coworkers.

#### 2.2.1. Synthesis of Pladienolide B by Kotake and Coworkers (2007)

**Scheme 1:**
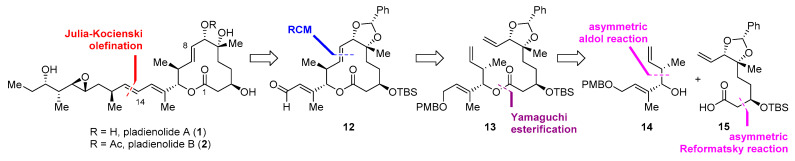
Kotake’s retrosynthetic analysis of pladienolides A and B (2007).

In 2007, the first total synthesis of pladienolide class natural products was reported by Kotake and coworkers [49]. Their approach for the construction of stereogenic centers is based on reagent-controlled stereoselective reactions to confirm the absolute configurations of the pladienolides. In their retrosynthetic plan (Scheme 1), the side chain unit and 12-membered macrolactone **12** of pladienolide B (**2**) could be disconnected by Julia–Kocienski olefination. Macrolactone **12** could be derived from **13** through RCM. Compound **13** was assembled by Yamaguchi esterification from fragments **14** and **15**, and both were prepared in an asymmetric manner by *anti*-aldol and Reformatsky reactions, respectively.

**Scheme 2:**
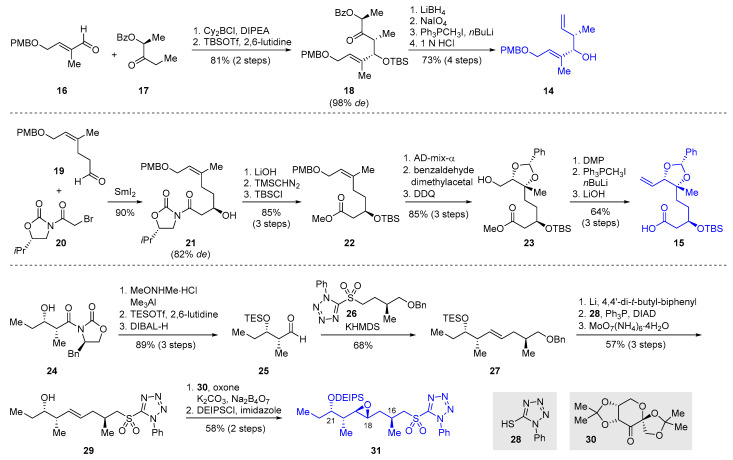
Preparation of three fragments for the synthesis of pladienolide B (Kotake, 2007).

The total synthesis commenced with the preparation of building blocks 14 and 15, as shown in Scheme 2. The absolute stereochemistry of **14** was established by Paterson *anti*-aldol condensation using ketone **17 [54]** as an external chiral source, where aldehyde **16** was converted to compound **18** with excellent diastereoselectivity (98% *de*) after TBS protection. The resulting aldol product was then transformed to fragment **14** in several steps, including the removal of benzoyloxy ketone and Wittig olefination of the resulting aldehyde. Another fragment, **15**, was also prepared in 10 steps from aldehyde **19** [55]. The Sm(II)-mediated asymmetric Reformatsky reaction of **19** with chiral auxiliary **20** afforded β-hydroxyamide **21,** with acceptable diastereoselectivity (82% *de*) [56]. The diastereomers were later separated with column chromatography on silica gel. Removal of the chiral auxiliary, methylation, and TBS protection allowed the formation of product **22**, which subsequently underwent asymmetric Sharpless dihydroxylation to afford compound **23,** after benzylidene acetal formation and PMB ether deprotection. The Dess–Martin oxidation of **23**, followed by the Wittig reaction and ester hydrolysis, finally provided key fragment **15**.

Side-chain fragment **31**, with five stereogenic centers at C16 and C18–21, was synthesized from the known *syn*-aldol product **24** [57]. Aldehyde **25** prepared from **24** through the Weinreb amide-mediated reduction was subjected to the Julia–Kocienski olefination with sulfone **26** to afford *trans*-olefin **27**. Benzyl deprotection, Mitsunobu reaction with **28**, and the resulting sulfide oxidation smoothly afforded sulfone **29**, which underwent asymmetric Shi epoxidation with chiral ketone **30** to stereoselectively yield side chain fragment **31**.

**Scheme 3:**
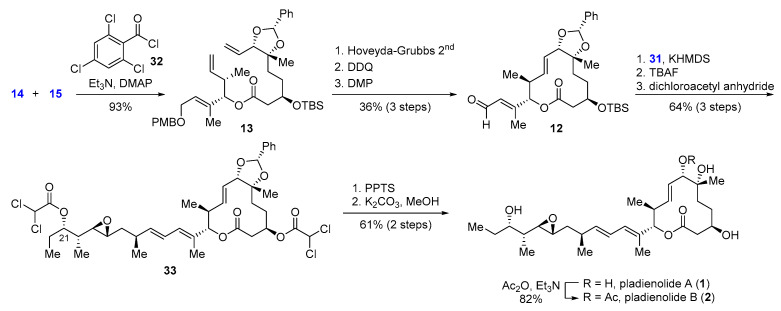
Completion of the total synthesis of pladienolides A and B (Kotake, 2007).

To complete the total synthesis, **14** was coupled with **15** under esterification conditions using Yamaguchi reagent **32**, leading to **13** in a 93% yield (Scheme 3). RCM, followed by a sequence of PMB deprotection/oxidation, afforded the macrocyclic aldehyde **12**. The Julia–Kocienski olefination between **12** and side-chain unit **31** afforded **33** upon silyl deprotection and reprotection of the resulting diol, with a dichloroacetyl group. Reprotection with the electron-deficient dichloroacetyl group was necessitated by the nucleophilic attack of the C21 hydroxy group on the proximal epoxide during benzylidene deprotection under acidic conditions. Finally, the removal of both benzylidene and dichloroacetyl groups provided pladienolide A (**1**), from which C7–OH was then selectively acetylated to yield pladienolide B (**2**), completing the first total syntheses of pladienolide-class natural products.

With the absolute structure of the macrocyclic core in hand, synthetic efforts were directed toward another natural anticancer analog, pladienolide D (**4**), whose structure differs from that of pladienolide B by one hydroxy group, located at C16. The absolute stereochemistry at C16 was confirmed to be *R* by the chemical degradation/derivatization of **4** and extensive NMR analysis. The conjugation of two fragments by the Julia–Kocienski olefination was expected to be ineffective in this case because of the presence of a quaternary C16. Thus, cross-metathesis (CM) was suggested to combine the fragments [58].

**Scheme 4:**
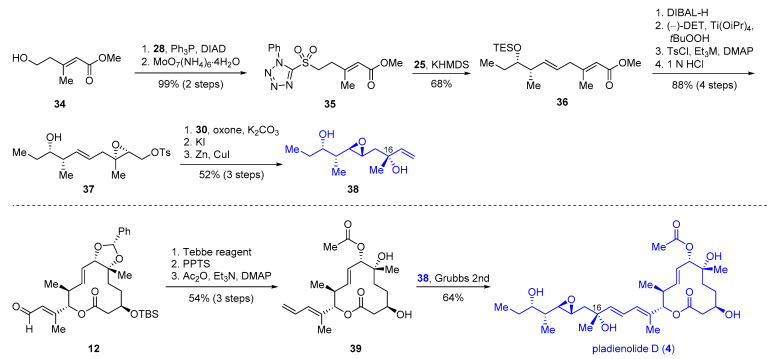
Preparation of the side chain fragment and completion of the total synthesis of pladienolide D (Kotake, 2007).

The synthesis of side-chain **38** commenced with the preparation of Julia–Kocienski reagent **35** from known alcohol **34** [59]. The olefination of **35** with **25** smoothly afforded *trans*-olefin **36**, which was successfully transformed to epoxide **37** by Sharpless asymmetric epoxidation, with moderate diastereoselectivity (90% *de*) (Scheme 4). The second epoxidation of **37** by Shi’s ketone **30**, followed by regioselective reductive epoxide cleavage, afforded the desired allylic alcohol **38**. Another compound required for olefin cross-metathesis, **39**, was obtained from **12** by sequential Tebbe olefination, global deprotection, and selective acylation. Finally, the cross-metathesis of **38** and **39** completed the first total synthesis of pladienolide D, with a 64% yield. All stereogenic centers in this synthesis were delivered and controlled with the aid of external chiral sources, such as chiral auxiliaries or chiral catalysis, to guarantee the absolute structure of complex natural products, despite the long linear steps required for this strategy.

#### 2.2.2. Synthesis of Pladienolide B by Ghosh and Anderson (2012)

**Scheme 5:**
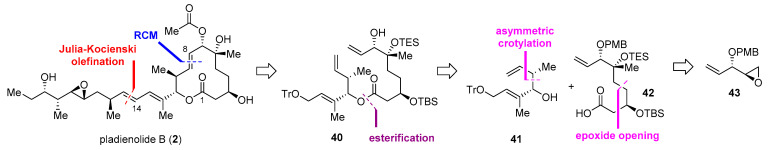
Ghosh and Anderson’s retrosynthetic analysis of pladienolide B (2012).

Ghosh and Anderson demonstrated a convergent synthetic pathway toward pladienolides to accelerate structural modification and structure–activity relationship studies (Scheme 5) [50]. Pladienolide B (**2**) was divided into small fragments with one or two stereogenic centers. Parallel to Kotake’s synthesis, the side chain unit could be connected to the core macrocycle by the Julia–Kocienski olefination, which could be accessed by an RCM of **40**. The formation of **40** could be achieved by the Yamaguchi esterification between building blocks **41** and **42**, which could be conveniently divided into small fragments, including **43**.

**Scheme 6:**
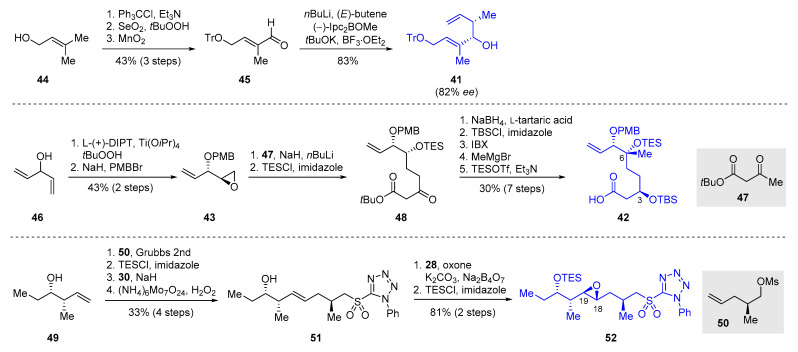
Preparation of the fragments for the synthesis of pladienolide B (Ghosh and Anderson, 2012).

First, building block **41** was prepared from prenol **44**, which was protected with a trityl group and oxidized to **45** (Scheme 6). Asymmetric Brown crotylation [60] furnished **41** with a moderate enantiomeric excess (82% *ee*). In parallel, the preparation of **42** began with Sharpless asymmetric epoxidation of commercially available divinyl carbinol **46**, which yielded **43** after PMB protection. β-Keto ester **47** was added to **43** to afford elongated chain **48**, which was subjected to a series of reactions, including asymmetric reduction with l-tartaric acid [61], and a substrate-controlled diastereoselective Grignard reaction to provide key building block **42** with two newly generated stereogenic centers at C3 and C6.

To synthesize side-chain unit **52**, Ghosh and Anderson employed cross-metathesis between two small fragments, namely, homoallylic alcohol **49** and mesylate **50**, whereas the Kotake group used the Julia–Kocienski olefination (Scheme 2). The *trans* selectivity in cross-metathesis was moderate, with an *E*/*Z* ratio of 5:1 (Scheme 6). Subsequent TES protection, the 1-phenyl-1H-tetrazole-5-thiol **28** substitution of mesylate, followed by sulfur oxidation, produced sulfone **51**. Asymmetric Shi epoxidation was also applied in this synthesis for the construction of epoxides at C18 and C19. TES protection completed the preparation of the Julia–Kocienski reagent **52**.

**Scheme 7:**
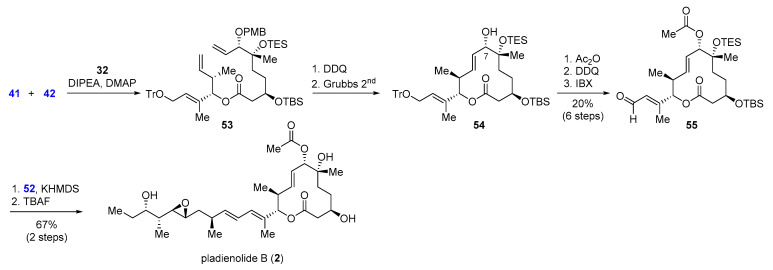
Final stage of the total synthesis of pladienolide B (**2**) (Ghosh and Anderson, 2012).

With all of these building blocks, an approach similar to Kotake’s synthesis was adopted to complete the synthesis of pladienolide B (Scheme 7). Fragments **41** and **42** were connected through the Yamaguchi esterification to afford **53**, which was macrocyclized by RCM to afford core **54**. After acetylation of C7–OH, deprotection of the trityl group and IBX oxidation furnished aldehyde **55**. Finally, the olefination of aldehyde **55** with sulfone **52** completed the total synthesis of pladienolide B. Ghosh and Anderson reported a convergent, scalable, and diversifiable synthesis involving 17 steps (LLS) and 1.4% overall yield.

#### 2.2.3. Synthesis of FD-895 by Burkart and Coworkers (2012)

**Scheme 8:**
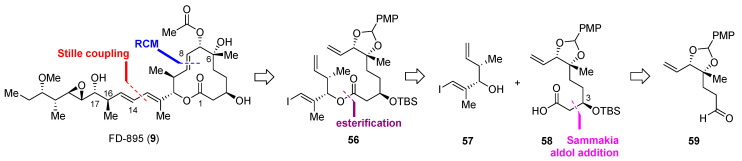
Burkart’s retrosynthetic analysis of FD-895 (2012).

Concurrent with Ghosh and Anderson’s synthesis (Scheme 5, Scheme 6 and Scheme 7), Burkart and coworkers completed the total synthesis of FD-895 (**9**), which shares the identical macrocyclic core of pladienolide B but has a more substituted side chain [34]. Before the isolation of pladienolides in 2004, FD-895 was isolated in 1994 from *Streptomyces hygroscopicus* A-9561 [33]. Through intensive and combinatorial NMR studies, the full structure was confirmed, except for the stereochemistry at C16 and C17 positions. To confirm the absolute configuration of FD-895, a retrosynthesis was designed by the Burkart group, providing four diastereomers at C16 and C17. The stereoisomeric side chains could be prepared in a stereodivergent manner and then assembled by Stille coupling with a macrocyclic core (Scheme 8). The core unit would be accessed by an RCM of **56**, which could be disconnected by esterification into two building blocks **57** and **58**. Asymmetric aldol addition of **59** was proposed, to provide **58** with a C3 stereogenic center.

**Scheme 9:**
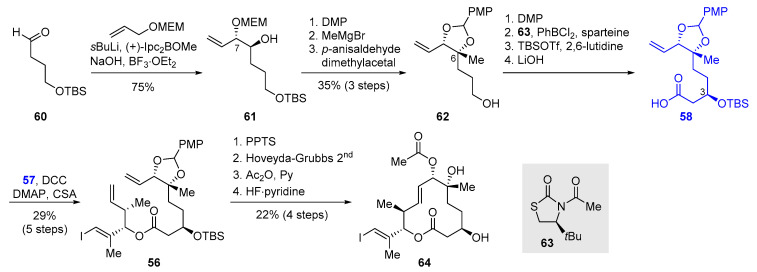
Synthesis of macrocyclic core **64** (Burkart, 2012).

As shown in Scheme 9, the synthesis of macrocyclic core **64** began with the asymmetric Brown allylation of aldehyde **60** for generation of the stereogenic center C7 in **61** [62]. The induction of stereogenicity at C6 of FD-895 (**9**) was guided by a C7 stereocenter using the chelate-controlled Grignard addition method [63]. The ketone resulting from the DMP oxidation of **61** was converted to carbinol **62** by chelate-controlled methylation and diol protection, with concomitant deprotection of TBS. To elongate the carbon frameworks, Sammakia aldol addition was employed, along with chiral auxiliary **63 [64]**, to afford esterification substrate **58** after TBS protection and hydrolytic cleavage. The known alcohol **57** was coupled with acid **58** to produce ester **56**, which was transformed into the 12-membered ring **64** via sequential reactions including RCM and acetylation. Although the synthetic strategy for the core scaffold was analogous to previous syntheses, Burkart and coworkers succeeded in shortening the purification steps.

**Scheme 10:**
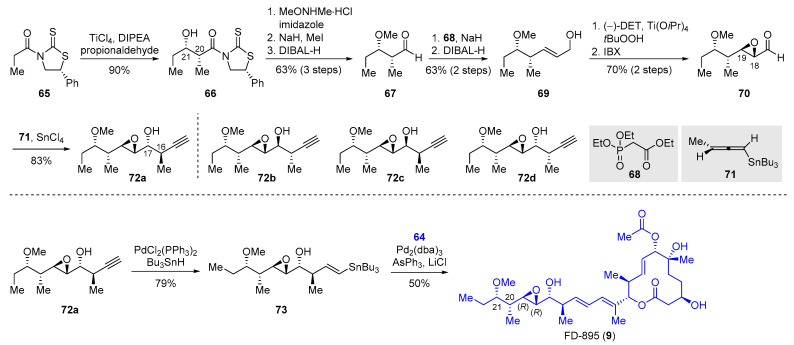
Final stage of the total synthesis of FD-895 (Burkart, 2012).

In the next stage, they prepared four plausible diastereomeric side chains in a divergent manner (Scheme 10). This divergent strategy was based on Marshall’s propargylation of allenylstannane from the common intermediate aldehyde **70** [65]. The *syn*-aldol product **66**, which contains two contiguous stereogenic centers (C20 and C21 of FD-895) was prepared from **65** using a method developed by Crimmins [66]. Conversion to the Weinreb amide, followed by methylation, and DIBAL-H reduction provided aldehyde **67**, which was immediately transformed to allyl alcohol **69** via the HWE olefination with **68** and reduction to minimize epimerization at C20. The C18–C19 epoxide was installed by Sharpless asymmetric epoxidation, and the subsequent oxidation with IBX produced the common intermediate **70** for the diastereomeric side chains. Marshall’s propargylation using diverse allenylstannanes allowed the divergent synthesis of **72a**–**72d**, in which **72a** with a 16*R*,17*R* configuration was diastereoselectively prepared from **71**.

In the final stage, hydrostannylation of the side chain isomers **72a**–**72d** provided Stille reagents **73a**–**73d**, which were utilized for cross-coupling reactions with macrocycle **64** to afford four isomers of FD-895 (**9**). As expected, the spectral data of one isomer (16*R*,17*R*) matched those of FD-895. Thus, Burkart and coworkers accomplished the first total synthesis of FD-895 and confirmed its absolute configuration at C16 and C17. Recently, a scalable synthetic method for 17*S*-FD-895 was also reported, which demonstrated anticancer activity 25 times stronger than that of FD-895 [67].

#### 2.2.4. Synthesis of Pladienolide B by Kumar and Chandrasekhar (2013)

**Scheme 11:**
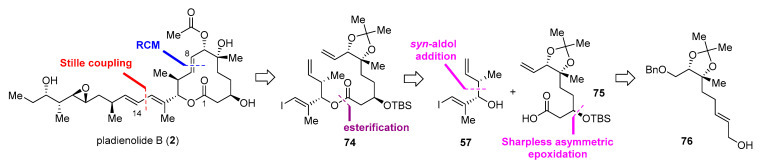
Kumar and Chandrasekhar’s retrosynthetic analysis of pladienolide B (2013).

When biologically meaningful natural products are discovered and applied to drug discovery, synthetic and medicinal chemists sometimes attempt to truncate the structure to simplify the complexity of the compounds involved. Thus, Kumar and Chandrasekhar reported the enantioselective synthesis of pladienolide B, as well as its side-chain-truncated analogs bearing simple aromatic groups, rather than the complex and linear chain of the natural product [51]. In their retrosynthetic analysis (Scheme 11), pladienolide B (**2**) could be disconnected by Pd-catalyzed Stille coupling into the side chain and the macrocyclic part, the latter of which would be available via RCM of **74**, similar to previous synthetic studies. Acid **75**, which can be readily connected with **57** to produce **74**, resulted from the oxidative adjustment of **76**.

**Scheme 12:**
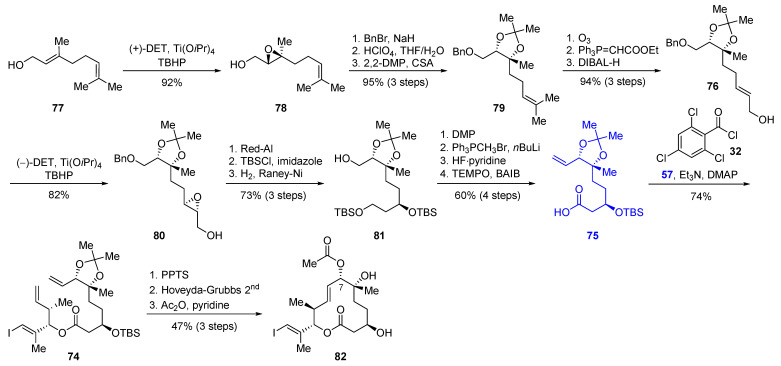
Construction of the macrocyclic core unit **82** (Kumar and Chandrasekhar, 2013).

As depicted in Scheme 12, the construction of the macrocyclic core **82** began with the Sharpless asymmetric epoxidation of geraniol (**77**) to afford *trans*-epoxide **78**. Benzyl protection and acid-mediated epoxide opening of **78** afforded **79** after diol protection [68]. Ozonolysis of trisubstituted olefin **79**, followed by HWE olefination and DIBAL-H reduction, provided allylic alcohol **76**, which underwent a second Sharpless asymmetric epoxidation to yield epoxy alcohol **80**. Reductive regioselective epoxide opening, directed by the adjacent alcohol, provided **81** after protection steps [69]. Oxidation of the resulting alcohol **81** produced the corresponding aldehyde, which allowed a series of reactions including the Wittig olefination to afford building block **75**. The connection of **75** with the known alcohol **57** was achieved under Yamaguchi conditions to afford **74**. While the RCM of **74** with Grubbs first- and second-generation catalysts was ineffective, the use of the Hoveyda–Grubbs second-generation catalyst instead afforded a moderate yield (52%) in the absence of the acetal group. Subsequent acetylation was carried out at a low temperature (−10 °C) to selectively introduce the acetyl group on C7–OH, to afford the cyclic core **82** in good yield (90%).

**Scheme 13:**
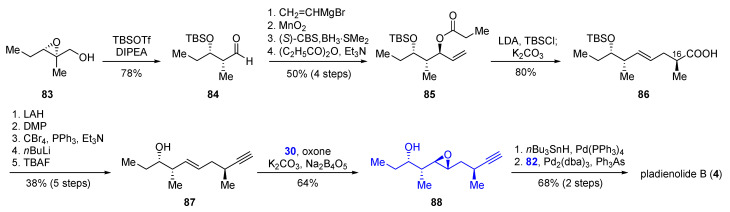
Final stage of the total synthesis of pladienolide B (Kumar and Chandrasekhar, 2013).

In the next stage, side-chain synthesis commenced with the distinctive hydride transfer of the known epoxy alcohol **83** (Scheme 13) [70]. Intramolecular hydride transfer in the presence of TBSOTf and the amine base regioselectively opened the epoxide to afford aldehyde **84**. Propionyl ester **85**, a substrate of the Ireland–Claisen rearrangement, was generated via Grignard addition, oxidation, CBS asymmetric reduction, and propionylation. Rearrangement under basic conditions was carried out to afford carboxylic acid **86**, delivering stereogenicity at C16 [71]. Further decorations, including aldehyde formation and the Corey–Fuchs reaction, provided alkyne **88** via **87**. Finally, the hydrostannylation of **88** and coupling of the resulting product with core unit **82** completed the total synthesis of pladienolide B (**4**) [72].

**Scheme 14:**
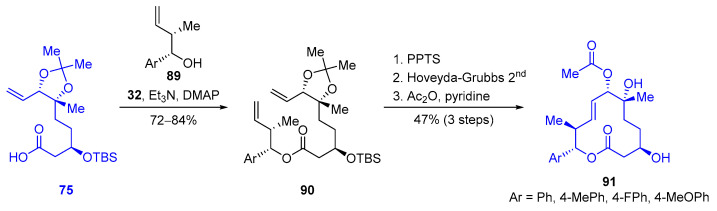
Synthesis of truncated pladienolide B analogs (Kumar and Chandrasekhar, 2013).

The versatility of the esterification–RCM sequence discussed in this section was demonstrated by Kumar and Chandrasekhar’s synthesis of the side-chain-truncated analogs **91** (Scheme 14). Coupling of advanced intermediate **75** with Evans *anti*-aldol products **89** afforded dienes **90**, which were converted to simplified pladienolide analogs **91**. They demonstrated that the truncated analog **91** demonstrates biological activities comparable to those of the natural product in the A549 cell line, indicating that the macrocyclic core plays a pivotal role in anticancer activity.

#### 2.2.5. Synthesis of 6-Deoxyoladienolide D by Keaney and Coworkers (2014)

**Scheme 15:**
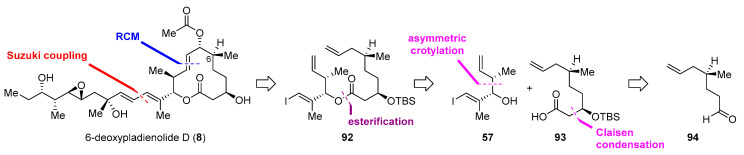
Keaney’s retrosynthetic analysis of 6-deoxypladienolide D (2014).

Keaney and coworkers at Eisai Co. reported the total synthesis of 6-deoxypladienolide D (**8**) in 2014, scarcely accessed by the semisynthetic pathway, despite its potent splicing inhibitory activity [52]. Rather than using precise and delicate synthetic strategies, they focused on scalable and industrial-friendly synthetic routes. From a retrosynthetic perspective, 6-deoxypladienolde D (**8**) is divided into the macrocyclic core unit and the diene chain, which can be combined by Suzuki coupling (Scheme 15). The 12-membered ring could be constructed by the RCM of ester **92**, which would be derived from **57** and **93** using a synthetic strategy akin to Burkart’s synthesis [34].

**Scheme 16:**
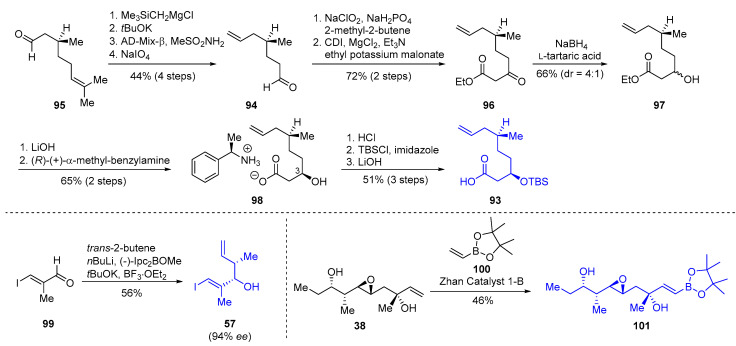
Total synthesis of 6-deoxypladienolide D (Keaney, 2014).

Preparation of building block **93** began with the commercially available citronellal (**95**) (Scheme 16). The Peterson olefination of the aldehyde group and oxidative cleavage of the olefin moiety produced aldehyde **94**. Pinnick oxidation, followed by Claisen condensation, yielded β-keto ester **96**, which underwent asymmetric reduction to afford diastereomeric mixture **97** with a ratio of 4:1. To cost-effectively isolate the pure diastereomer, a chiral resolution of **97** was achieved with (*R*)-(+)-α-methyl-benzylamine. The stereochemistry at C3 of **98** was confirmed by X-ray crystallography. Building block **93** was then obtained via a three-step sequence: acidification, silylation, and hydrolysis. One of the key building blocks in pladienolide synthesis, **57**, was generated on a bulk scale by the Brown asymmetric crotylation of aldehyde **99** [73]. Additionally, the synthesis of the side-chain counterpart **101** utilized cross-metathesis between Kotake’s intermediate **38** [49] and vinyl boronate **100**.

**Scheme 17:**
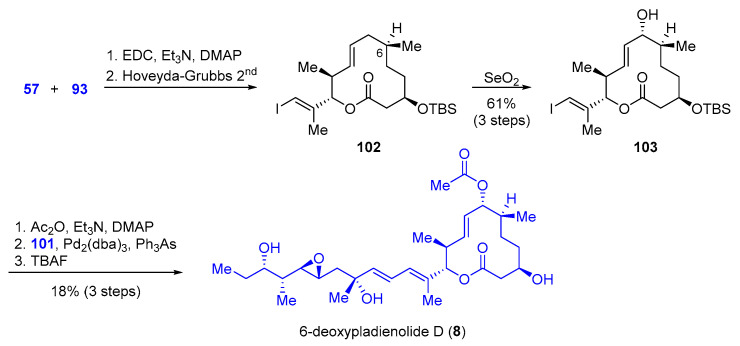
Final stage of the total synthesis of 6-deoxypladienolide D (Keaney, 2014).

The final stage of the macrolide synthesis proceeded according to general synthetic procedures, including esterification of the two building blocks and RCM (Scheme 17). Allylic oxidation of the corresponding macrocycle **102**, the key reaction in this synthesis, was preliminarily simulated via computational modeling. Influenced by the C6-methyl group, stereoselective oxidation at the C7 position was predicted and realized in moderate yield and complete diastereoselectivity. Sequential acetylation of **103**, Suzuki coupling with **101**, and TBS deprotection completed the total synthesis of 6-deoxypladienolide D (**8**). Keaney’s synthesis requires the use of inexpensive commercially available starting materials and late-stage functionalization to provide a sufficient quantity of scarce 6-deoxypladienolide B, to confirm the biological activity against mutant SF3b1.

#### 2.2.6. Synthesis of Pladeinolide B by Yoo and Krische (2021)

**Scheme 18:**
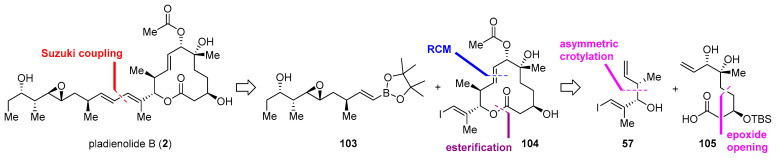
Yoo and Krische’s retrosynthetic analysis of pladienolide B (2021).

Given the continuous demand for efficient and practical synthesis of complex pladienolide-type natural products, which have more than 11 stereogenic centers, Yoo and Krische designed a remarkably concise convergent synthesis of pladienolide B (**2**) [53]. By utilizing the state-of-the-art synthetic methodology developed by themselves, key fragments for the convergent synthesis were expected to be prepared [74,75]. As anticipated, the total synthesis of pladienolide B has been finished in only 10 longest-linear steps. Most stereogenic centers on the building blocks **103**, **57**, and **105** were established by their unique synthetic methods (Scheme 18).

**Scheme 19:**
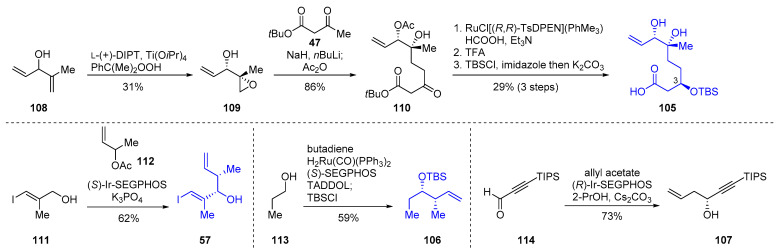
Preparation of fragments required for the synthesis of pladienolide B (Yoo and Krische, 2021).

Yoo and Krische’s synthesis is based on the parallel preparation of small fragments **57**, **105**, **106**, and **107** (Scheme 19). Preparation of **105** commenced with the Sharpless asymmetric epoxidation of allylic alcohol **108** [76]. Regioselective epoxidation of more substituted alkenes in **108** afforded the highly enantiomerically rich epoxide **109** (dr = 20:1, 95% *ee*), which was regioselectively opened by the dienolate **47** to afford **110** after one-pot acetylation. Noyori asymmetric hydrogenation of **110** generated a stereogenic center at C3 with a moderate selectivity of 4:1. Protecting group adjustment successfully provided building block **105**. The remaining building blocks were prepared via hydrogenative asymmetric crotylation. Allylic alcohol **57** was provided in a one-step reaction: Ir-catalyzed alcohol-mediated *anti*-crotylation of **111** and **112**. Building blocks of the side-chain fragments **106** and **107** were also accessed by Ru-catalyzed *syn*-crotylation and Ir-catalyzed asymmetric allylation of **113** and **114**, respectively. 

**Scheme 20:**
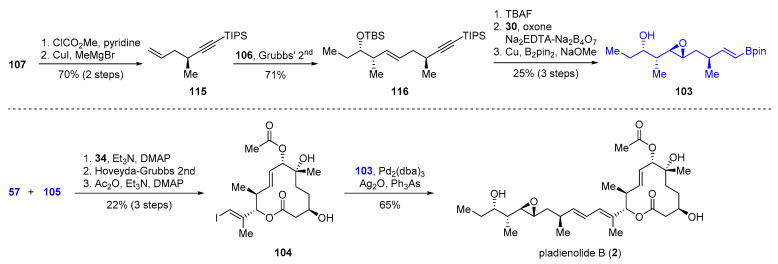
The completion of the total synthesis of pladienolide B (Yoo and Krische, 2021).

After substitution of the hydroxy functional group in **107** with a methyl group under the Normant condition [77], cross-metathesis between the resulting alkynes **115** and **106** was conducted to provide the side chain carbon skeleton **116** (Scheme 20) [78]. Compound **116** was transformed to Suzuki reagent **103** via silyl deprotection, Shi epoxidation, and hydroboration [79]. In the final stage, Suzuki coupling of **103** and the core unit **104**, prepared from **57** and **105** by Yamaguchi esterification and RCM, completed the total synthesis of pladienolide B (**2**). Yoo and Krische demonstrated the shortest stereodivergent synthetic routes for the pladienolide series by maximizing the usefulness of their distinctive chemistry. 

### 2.3. Synthesis via Macrolactonization

Another strategy to construct the 12-membered macrocyclic pladienolide core is macrolactonization. Because macrocyclic lactone moieties are abundant in natural substances (e.g., 8-membered octalactins and 60-membered quinolidomicins) and natural macrocyclic lactones have exhibited a wide range of interesting properties such as medicinal/insecticide activity, fragrance production, and phytotoxicity, synthetic approaches have been extensively studied, particularly in the field of natural product synthesis [80]. Three syntheses of pladienolide B or its core structure that use macrolactonization have been reported by Skaanderup and Jensen [81], Maier and coworkers [82,83], and Rhoades et al. [84]. 

#### 2.3.1. Synthesis of the Macrocyclic Core of *ent*-Pladienolide B by Skaanderup and Jensen (2008)

**Figure 4:**
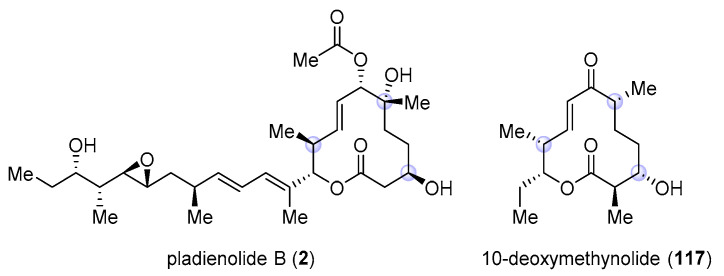
Structural comparison of pladienolide B (**2**) and 10-deoxymethynolide (**117**).

At the outset of the study by Skaanderup and Jensen (2008), the absolute and relative stereochemistry of pladienolides had not been completely revealed. This allowed them to synthesize the *ent*-pladienolide B core structure devoid of the side chain, because the structurally similar polyketide, 10-deoxymethynolide (**117**, Figure 4), was previously reported to have similar enantiomeric stereochemistry to that of pladienolide B.

**Scheme 21:**
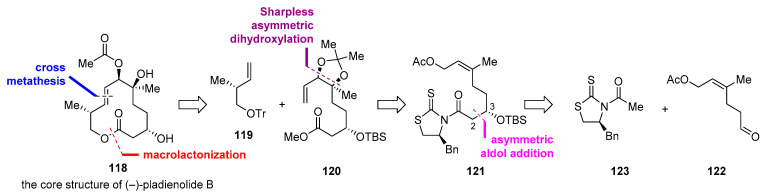
Skaanderup and Jensen’s retrosynthetic analysis (2008).

As outlined in Scheme 21, the core structure of the *ent*-pladienolide B **118** could be accessed by macrolactonization to form a 12-membered ring, the linear substrate of which is conceivably disconnected by cross-metathesis into **119** and **120**. The vicinal diol moiety **120** and its relative stereochemistry were built up by the Sharpless asymmetric dihydroxylation of **121**. The C2–C3 bond formation is achievable via the asymmetric aldol reaction of aldehyde **122,** with the aid of a chiral auxiliary **123**.

**Scheme 22:**
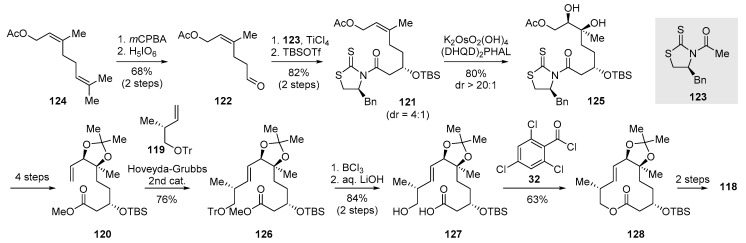
Synthesis of the core structure of *ent*-pladienolide B (Skaanderup and Jensen, 2008).

The synthesis of core structure **118** by Skaanderup and Jensen began with commercially available acetate **124**, which underwent selective epoxidation followed by oxidative cleavage to produce aldehyde **122** (Scheme 22). Chiral acetylthiazolidinethione **123** was then employed for asymmetric aldol addition with **122** to yield **121**, albeit with a somewhat low diastereomeric ratio (dr = 4:1). After Sharpless asymmetric dihydroxylation to convert **121** to diol **125**, the detachment of the chiral auxiliary, followed by Wittig methylenation, yielded a cross-metathesis substrate **120**. Subsequent Hoveyda–Grubbs catalyst-mediated cross-coupling with homoallyl ether **119** afforded product **126** in moderate yield. Selective deprotection of the trityl ether in the presence of an acetal protecting group produced *seco*-acid **127** after methyl ester hydrolysis. Fine-tuning of the Yamaguchi condition eventually furnished 12-membered cycle **128**, which was then modified to the core structure of *ent*-pladienolide B. This is the first example of access to a 12-membered ring via macrolactonization as the last key step, and core structure **118** was obtained in 15 steps with an overall yield of 10.0%. 

#### 2.3.2. Synthesis of Pladienolide B by Maier and Coworkers (2014)

**Scheme 23:**
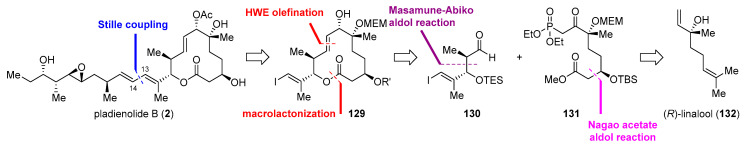
Maier’s retrosynthetic analysis of pladienolide B (2014).

In 2014, Maier and coworkers described a total synthesis of pladienolide B (**2**), incorporating the Horner–Wadsworth–Emmons olefination/macrolactonization sequence as key chemistry for the formation of 12-membered core **129** (Scheme 23) [82]. The precursors of **129**, fragments **130** and **131**, could be prepared by asymmetric aldol reactions, establishing their absolute and relative stereochemistry. The main skeleton of **131** was constructed by several transformations of commercially available (*R*)-linalool (**132**).

**Scheme 24:**
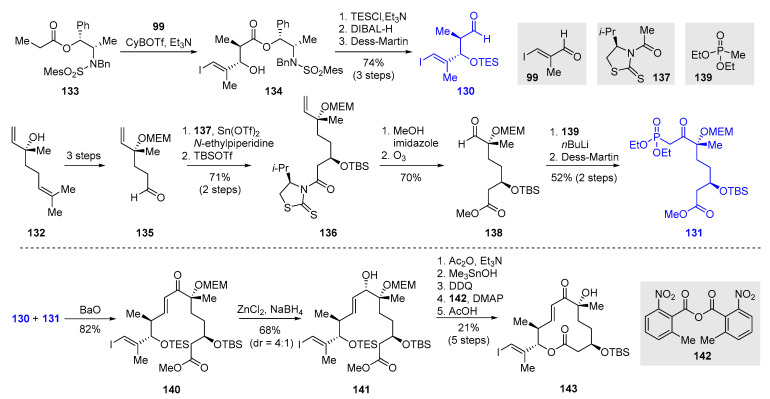
Synthesis of the macrocyclic core unit **143** (Maier, 2014).

First, aldehyde fragment **130** with *anti*-stereochemistry was prepared by the Masamune–Abiko aldol reaction protocol using chiral ester **133** (Scheme 24) [85,86], after chiral auxiliary detachment and oxidation [83]. The synthesis of fragment **131** was carried out in parallel with (*R*)-linalool (**132**). Three steps, including the oxidative cleavage of the trisubstituted olefin, afforded **135**, which was converted to **136** via the Nagao acetate aldol reaction with chiral methyl ketone **137 [87,88,89]**. Following the establishment of the required stereochemistry, the chiral auxiliary was removed from compound **136,** and the resulting compound was treated with **139** to introduce the methylphosphonate group, affording the HWE reaction substrate **131**. Aldehyde **130** was then combined with methylphosphonate **131** using an HWE reaction to furnish elongated enone **140**, which was reduced to produce **141** by a chelation-controlled reduction with a moderate diastereomeric ratio. Through several transformations, including Shiina-type macrolactonization using 2-methyl-6-nitrobenzoic anhydride (**142**) [90], the synthesis of the metal-catalyzed reaction substrate **143** was accomplished.

**Scheme 25:**
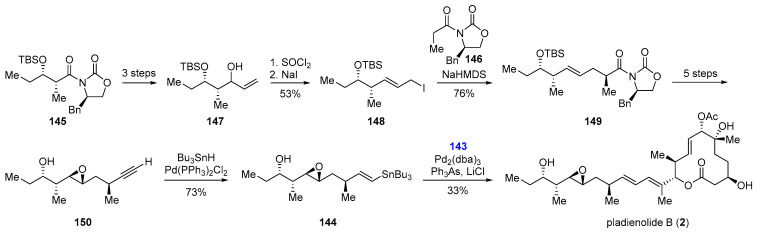
Preparation of the side-chain unit **144** and the final stage of the total synthesis of pladienolide B (Maier, 2014).

The required side chain, **144**, was prepared, as shown in Scheme 25. The known TBS-protected aldol product **145 [57,91]**, produced using **146**, was converted to allyl alcohol **147**, which was then subjected to Sn2′ chlorination and the Finkelstein reaction, which yielded allyl iodide **148**. The second employment of **146** for asymmetric allylation furnished **149**, which underwent the redox sequence, Shi epoxidation, and Seyferth–Gilbert homologation to provide terminal alkyne **150**. Alkyne **150** was subjected to palladium-catalyzed hydrostannylation to afford another counterpart **144**, which, upon subsequent Stille coupling with **143**, finally furnished pladienolide B (**2**). This total synthesis was completed in 18 steps (LLS) and 0.6% overall yield.

#### 2.3.3. Total Synthesis of Pladienolide A, B, and H3B-8800 by Rhoades et al. (2021)

**Scheme 26:**
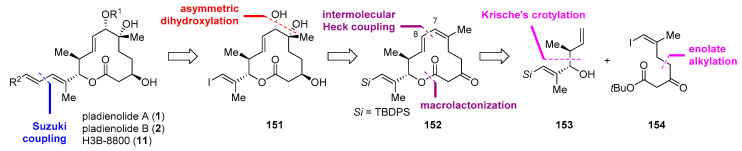
Rhoades et al.’s retrosynthetic analysis of pladienolide A, B, and H3B-8800 (2021).

Rhoades et al. completed the total synthesis of pladienolides A and B as well as the synthetic analog H3B-8800 (**13**), which are currently under phase 1 clinical trials [84]. Their asymmetric synthesis featured a protecting group and chiral auxiliary-free approaches, resulting in 10-step (LLS)-total synthesis from commercially available building blocks. Retrosynthetically, the formation of pladienolide class products **1**, **2**, and **11** divergently results from vinyl iodide **151**, via Suzuki coupling with diverse vinyl borane counterparts (Scheme 26). The diol moiety of **151** was introduced from **152** under the control of the site- and stereoselectivity. The 12-membered ring of **152** could be accessed by macrolactonization after the intermolecular Heck reaction between terminal olefin **153** and vinyl iodide **154** forming a C7–C8 bond with geometrical selectivity. The absolute and relative stereochemistry of homoallyl alcohol **153**, identical to those of natural products, could be established via Krische’s crotylation protocol.

**Scheme 27:**
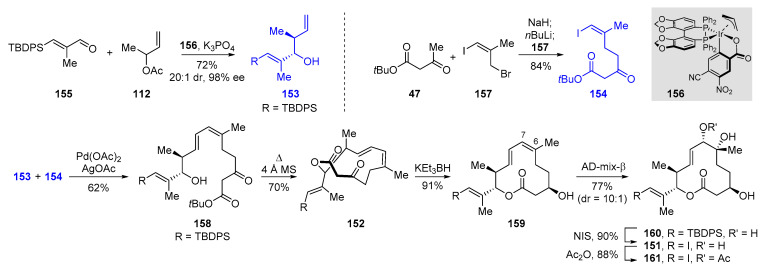
Preparation of a 12-membered macrocyclic unit (Rhoades et al., 2021).

Building blocks **153** and **154,** Heck coupling substrates, were prepared efficiently as shown in Scheme 27. Initially, the terminal olefin **153** was synthesized from enal **155**, which can be obtained from commercially available propyne in two steps [92] and 3-buten-2-yl acetate **112** by Krische’s crotylation method [93] using a chiral Ir catalyst **156** in excellent diastereomeric and enantiomeric ratios. Dianion formation of β-keto ester **42**, followed by the addition of allyl bromide **157**, afforded vinyl iodide **154**. Heck coupling between **153** and **154** in the presence of silver(I) salts afforded (*E*)-**158** with (*Z*)-**158** as a minor product (*E*:*Z* = 7:3). Heating **158** in toluene was effective in producing macrolactone **152**, which was converted to alcohol **159** using stereoselective ketone reduction directed from outside the ring system. The subjection of **159** to the Sharpless asymmetric dihydroxylation with the use of AD-mix-β yielded triol **160** with good diastereoselectivity (dr = 10:1) and excellent regioselectivity toward the C6–C7 olefin despite the presence of other olefins. NIS-mediated silicon-iodine exchange [94] provided Suzuki reaction substrate **161** after selective acetylation of the 7-OH of **151**.

**Scheme 28:**
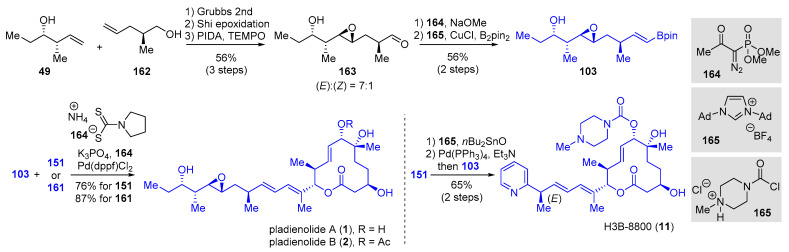
Final stage of the total synthesis of pladienolide A, B, and H3B-8800 (Rhoades et al., 2021).

Side-chain unit **103**, a Suzuki coupling counterpart, was prepared by a cross-metathesis–alkyne hydroboration strategy, which was also adopted in Yoo and Krische’s synthesis (Scheme 28). Further, **103** was elongated by cross-metathesis between **49** and **162** using the Grubbs 2nd generation catalyst. Shi epoxidation of the corresponding internal olefin and primary alcohol oxidation afforded **163**, the aldehyde of which was converted to a terminal alkyne with Bestmann–Ohira reagent (**164**). Hydroboration with *N*-heterocyclic carbene ligand **165** was effective in delivering the vinyl borane group of **103** [95]. The natural products pladienolide A (**1**) and B (**2**) were obtained by the Suzuki coupling of **103** with **151** and **161**, respectively. The use of **164** during the workup resulted in the efficient removal of palladium metals [96]. For the synthesis of H3B-8800 (**11**), the core unit **151** was reacted with **165** in the presence of dibutyltin oxide to produce the carbamate, which upon Suzuki reaction with **103**, was finally transformed to **11**. It is the shortest synthesis of H3B-8800 (10 LLS from commercially available material, with 10.1% yield; compared with Eisai Co.: 12 LLS, 4.2% overall yield) [97].

## 3. Conclusions

This review focused on the total syntheses of pladienolide-class natural products that exhibit spliceosome-modulating activity by binding to the SF3b unit. These polyketides have become an attractive target for the synthetic community because of the synthetic challenges afforded by its 12-membered macrocyclic framework, as well as up to 10 stereogenic centers. Total syntheses for compounds of this class to date can be divided into two groups, according to the key method used to access the macrocyclic unit—RCM or macrolactonization. The yields of both cyclization steps are moderate to excellent (51–93%) as indicated in Table 1. The overall yields of LLS are also shown in Table 1, which reveals that Rhoades et al. completed the total synthesis of pladienolides with the best overall yield (ca. 12%) by virtue of the shortest steps (10 or 11 steps). 

The synthetic feature of each synthesis is summarized in Table 2. The syntheses utilizing RCM reaction commonly share intermolecular esterification–RCM sequence for the construction of the macrocyclic core. On the other hand, macrolactonization-mediated syntheses utilize each different sequence for preparing macrocyclic cores. Most stereocenters on pladienolides were established in a reagent-controlled manner. The stereogenic methyl and hydroxy groups on both the macrocyclic core and the side chain fragment are mostly controlled by an asymmetric aldol reaction using a chiral auxiliary. The epoxide in the chain was installed using external chiral sources. Conjugation between the two fragments was conducted by metal-catalyzed coupling or olefination. These synthetic efforts finally elaborated the bioactive synthetic analogs, E7107 and H3B-8800, the latter of which is currently under phase 1 clinical trials to treat patients with various hematologic malignancies. Given the increasing interest in RNA splicing for drug discovery, as well as the promising potential as anti-cancer agents, the rapid development of synthetic and medicinal chemistry leveraging pladienolide-class natural products and their analogs is anticipated in the near future.

## Figures and Tables

**Table 1:** Summary of total syntheses of pladienolides.

Cyclization Method	Year	Group	Synthetic Target	Cycliz-ation Yield	LLS	Overall Yield (LLS)	Scheme	Refs.
RCM	2007	Kotake	pladienolide A	93%	21	3.5%	1–4	[49]
			pladienolide B		22	2.9%		
			pladienolide D		20	3.1%		
	2012	Ghosh	pladienolide B	83%	17	1.4%	5–7	[50]
	2012	Burkart	FD-895	48%	15	0.8%	8–10	[34]
	2013	Chandrasekhar	pladienolide B	52%	21	7.0%	11–14	[51]
	2014	Keaney	6-deoxypladienolide D	77%	18	0.6%	15, 16	[52]
	2021	Krische	pladienolide B	51%	10	0.8%	18–20	[53]
Macrolactonization	2008	Skaanderup	macrocycle of pladienolide B	63%	15	10.0%	21, 22	[81]
	2014	Maier	pladienolide B	93%	17	2.0%	23–25	[82,83]
	2021	Rhoades–O’Malley–Wang	pladienolide A	70%	9	11.8%	26–28	[84]
			pladienolide B		10	11.9%		
			H3B-8800		10	10.1%		

**Table 2:** Synthetic features of the total syntheses covered in this review.

Cyclization Method	Group	Synthetic Features
RCM	Kotake (Section 2.2.1)	✓ First total synthesis of pladienolide natural products ✓ Confirmation of the absolute configuration of pladienolides ✓ Reagents-controlled stereoselective reactions
	Ghosh (Section 2.2.2)	✓ Convergent and scalable synthesis of pladienolides ✓ Convergency enabling convenient access to various derivatives
	Burkart (Section 2.2.3)	✓ First total synthesis and structure confirmation of FD-895 ✓ Stereodivergent synthesis of side chains to confirm the absolute structure ✓ First synthetic approach utilizing cross-coupling to append the side chain unit
	Chandrasekhar (Section 2.2.4)	✓ Synthetic flexibility provides side-chain truncated analogs ✓ Side-chain truncated analogs prove the importance of the macrocyclic core
	Keaney (Section 2.2.5)	✓ Industrial-friendly and cost-effective synthetic approach ✓ Chemoselective allylic oxidation to generate C-7 hydroxy group ✓ User-friendly Suzuki coupling reaction for the introduction of side chains
	Krische (Section 2.2.6)	✓ The shortest total synthesis of pladienolide B ✓ Metal-mediated asymmetric dehydrogenative C-C coupling ✓ Catalyst-oriented asymmetric synthesis maximizing synthetic convergence
Macro-lactonization	Skaanderup (Section 2.3.1)	✓ First synthetic example of the enantiomer of pladienolide B ✓ Cross-metathesis–macrolactonization sequence employed ✓ The use of Yamaguchi reagent for macrolactonizations
	Maier (Section 2.3.2)	✓ The use of commercially available chiral substance at the initial stage ✓ HWE olefination–macrolactonization sequence employed ✓ Employment of Shiina-type macrolactonization condition
	Rhoades–O’Malley–Wang (Section 2.3.3)	✓ The shortest total synthesis of H3B-8800, pladienolide A and B ✓ Heck coupling–macrolactonzation sequence employed ✓ Thermal macrolactonization conducted in the absence of coupling reagents
